# Recurrent Evans Syndrome in a Patient With 22q11.2 Deletion Syndrome: An Uncommon Hematological Presentation

**DOI:** 10.7759/cureus.11510

**Published:** 2020-11-16

**Authors:** Hector A Oliveras-Cordero, Enid Rivera-Jiménez

**Affiliations:** 1 Internal Medicine - Pediatrics, University of Puerto Rico, Medical Sciences Campus, San Juan, PRI; 2 Hematology and Oncology, University of Puerto Rico, Medical Sciences Campus, San Juan, PRI

**Keywords:** evans syndrome, 22q11.2 deletion syndrome, immune disorder, aiha, itp, immune dysregulation

## Abstract

We discuss the case of a three-year-old female patient who presented with a severe episode of immune thrombocytopenia (ITP) and autoimmune hemolytic anemia (AIHA), confirming a diagnosis of Evans syndrome (ES). Over time, she continued to have several episodes of recurrent ITP until, several years later, she experienced a recurrent severe, refractory ES episode. Initially, she responded well to conventional treatment with steroids and intravenous immunoglobulin (IVIG); however, during later episodes, she required anti-CD20 therapy (rituximab). Due to peculiar facies and severe clinical presentation, an underlying immune dysregulation was suspected, which was later confirmed to be 22q11.2 deletion syndrome (22q11.2DS). Over time, her baseline immunoglobulin production decreased significantly. After monthly IVIG replacement, she had a marked reduction in ITP or AIHA events. 22q11.2DS is a frequently underdiagnosed primary immune disorder (PID). Low immunoglobulin production and recurrent ES are infrequent events associated with 22q11.2DS. This condition might cause profound immune dysregulation, predisposing patients to immune-related hematological dyscrasias that still need further research to be fully understood and characterized. We describe a case of 22q11.2DS and recurrent ES episodes, which involves a 13-year history of longitudinal follow-up care.

## Introduction

22q11.2 deletion syndrome (22q11.2DS) is a genetic condition with extremely variable phenotypes that include DiGeorge syndrome, conotruncal cardiac anomalies, and velocardiofacial syndrome, among other disorders. Its incidence has been estimated to be in the range of one in 2,000 to one in 4,000 newborns. Absent or hypoplastic thymus, cardiac anomalies, hypocalcemia, and parathyroid hypoplasia are the most classic findings associated with this condition. A primary cellular immunodeficiency due to thymic hypoplasia has also been classically reported [1-3]. More recent studies have shown that they can present with secondary antibody-mediated immunodeficiencies and with combined autoimmune hematological dyscrasias. This case is an example of the latter, and in this report, we intend to bring to light how those discrete immunodeficiencies seen in 22q11.2DS may interact and increase the risk of severe immune-mediated disorders in these patients.

## Case presentation

The patient was a female born at 39 weeks of gestational age by C-section (C/S) due to maternal history of previous C/S. Her birth weight had been 3.18 kg (45th percentile). She had been born without major complications but had been admitted to the neonatal intensive care unit (NICU) for cardiologic evaluation for a 3/6 holosystolic murmur. A ventricular septal defect (VSD) had been found without hemodynamic compromise. At two months of age, she had been found with congenital hip dislocation. During early infancy, both motor and speech delays had been identified. She had not had any history of recurrent infections in early infancy.

At three years of age, she presented with several days of excessive and prolonged gum bleeding, hematomas, and petechiae. She had been found to have severe thrombocytopenia and transferred to our institution. The patient had a history of an upper respiratory tract infection two to three weeks before evaluation. Physical exam showed generalized petechiae, frontal and submental hematoma, and scattered healing bruises. She was also found to have dysmorphic facies including frontal bossing, dental non-occlusion, and a short, webbed neck. The spleen was palpable at 2 cm below the costal margin. No palatal or renal abnormalities were found. Besides severe thrombocytopenia, she also had mild anemia and leukopenia. A bone marrow aspiration reported normal cellularity, flow cytometry, and cytogenetics. Over the next 48 hours, the leukopenia resolved, but the anemia dropped to 7 gm/dl with normal lactate dehydrogenase (LDH) and bilirubin levels. A direct antiglobulin test (DAT) was reported positive, finding anti-small-e antibodies. The confirmation of autoimmune hemolytic anemia (AIHA), on top of immune thrombocytopenia (ITP), prompted a reclassification of her clinical problem to Evans syndrome (ES). Baseline immunoglobulin levels were reported as normal to low-normal levels. Hepatitis panel, cytomegalovirus (CMV), Epstein-Barr virus (EBV), and human immunodeficiency virus (HIV) serologies were negative. The patient was treated with steroids and IVIG with a very good response. A genetic evaluation generated suspicion of Noonan syndrome. Over the next three years, the patient was lost to follow-up and received treatment several times with steroids due to recurrent ITP at another institution.

At the age of seven years, she presented again to our institution with hypoactivity and active bleeding from the oral and nasal mucosa. Laboratory workup showed severe thrombocytopenia and Coombs-positive hemolytic anemia, confirming recurrent ES. She was found to have a worsened case of hypogammaglobulinemia. Being refractory to steroids and IVIG, she only responded to anti-CD20 therapy. A fluorescence in situ hybridization test showed 22q11.2 deletion.

Over the next 10 years, she developed profound persistent hypogammaglobulinemia with most measurements showing immunoglobulin G (IgG) levels between 150-250 mg/dL, for which she received monthly immunoglobulin replacement. She has had occasional ITP relapses, but no further episodes of AIHA have been reported. During adolescence, she developed severe thoracic scoliosis that required spinal fusion and insertion of Harrington rods. She has had chronic iron deficiency related to poor iron intake due to oral hypersensitivity to solid foods and pills. She also developed severe vitamin B12 deficiency without diarrhea, macrocytosis, parietal cells, or intrinsic factor antibodies, with normal homocysteine levels. She continues to be managed with IV iron replacement and oral B12 supplementation. She is being trained in activities for daily living at a special educational institution, due to her moderate intellectual disability and autism spectrum disorder profile. The patient has not experienced any recurrent infections or hospitalizations in the last five years.

## Discussion

ES is characterized by the simultaneous or sequential development of immune thrombocytopenic purpura and AIHA. It can be the first manifestation of underlying immunodeficiencies. Some review articles have been published to explain the mechanisms that cause immunological system dysregulation/deficiency to be the possible catalyst for autoimmune cytopenias [1]. Reports in the literature suggest that up to 50% of cases of ES may be associated with autoimmune disease, lymphoproliferative disease, or primary immunodeficiencies.

Early childhood onset of severe ES is an uncommon hematological presentation of the 22q11.2DS spectrum, although immunodeficiency affects up to 75% of pediatric patients with this disorder [4]. Epidemiologic studies of ES in childhood are scarce, with some studies reporting an incidence that ranges between 0.5 and 1.2/1,000,000 person-years [5]. In the past, several cases have reported distinct hematological abnormalities in patients with this syndrome, mostly related to immune dysregulation that arises primarily from the thymic hypoplasia and T-cell dysfunction described in the syndrome. However, profound T-cell dysfunction may precipitate further immune dyscrasias by means of an alteration in T-cell-mediated B-cell function and regulation [1,3].

Thrombocytopenia presents in several ways as a related hematological finding in patients with 22q11.2DS [4-10]. One study has reported the prevalence of thrombocytopenia and large platelets in 22q11.2DS to be 35% and 82%, respectively [7]. This finding could be an isolated phenotypic presentation secondary to the established correlation between the loss of the gene encoding for the glycoprotein Ib platelet subunit beta protein, also in chromosome 22. This protein is part of the complex that mediates both platelet adhesion and aggregation, and its mutation is known to cause Bernard-Soulier syndrome [8-11]. However, another possible etiology for thrombocytopenia in 22q11.2DS would be autoimmune destruction of platelets as seen in ITP. Some reports have been published with findings similar to that of our patient who primarily presented with ITP [2]. The mechanism of developing ITP may be related to the defective T-cell function by dysfunctional effectors against auto-reactive clones with T-cell receptors directed against self-antigens. T-cell defects may also lead to faulty T-cell-B-cell interactions, which have deleterious effects on the B-cell maturation and regulation and hence may predispose to autoimmunity [1].

Other reports have included the discussion of AIHA as isolated or associated with other cytopenias. Sakamoto et al. reported isolated AIHA in a patient with 22q11.2DS who presented with pallor and hypoactivity. This patient had an increased reticulocyte count, a positive DAT, and normal leukocyte and platelet count. As part of their discussion, they emphasize the importance of follow-up in these patients. They explain that isolated dyscrasias may only be the first hematologic manifestation of a number of further hematologic complications [5,11,12]. The possible etiologies of AIHA are the same immunologic defects and interactions described above.

Our patient also had persistent progressive hypogammaglobulinemia, which has been described in patients with DiGeorge syndrome with a prevalence of up to 6%. This complication is most probably secondary to faulty production or faulty T-cell function and its impact on B-cell regulation and thus humoral immunity [12].

The pathophysiology of cytopenias in 22q11.2DS may be more complex than previously thought, as standard classification may fail to grasp all the cellular interactions that may be causing them. The primary immunodeficiency in 22q11.2DS is T-cell dysfunction, which may present with autoimmune T-cells and decrease cellular-mediated immunity regulation. An example of secondary immunodeficiency seen in 22q11.2DS is represented by hypogammaglobulinemia, as described above, which is an example of antibody-mediated autoimmunity as seen in ES. Lastly, the interaction between these two types of immune deficiencies may play a part in dysregulating the overall immune system and may even be the factor that ultimately is posing an increased risk for these patients to develop autoimmune cytopenias [13-15].

## Conclusions

This case emphasizes the importance of further exploration and characterization of the hematological presentations secondary to the immune dysregulation pathways that have been reported in 22q11.2DS as well as other disorders of immunodeficiency. The variability of phenotypic presentation in patients with 22q11.2DS makes it particularly difficult to establish clinical correlations as they can present with a vast array of both cellular and humoral deficiencies. Currently, the exact molecular interactions that increase the risk in these patients to develop immune-related hematological dyscrasias are not well understood. Further investigations of 22q11.2DS may eventually reveal the exact defective steps in the crosstalk pathways between T-cell and humoral immunity. Future studies should try to explain why they have an increased risk for developing autoimmune complications and propose possible means to treat said complications. Advancing our understanding of these immune presentations will eventually lead to improved care for these pediatrics patients, which will require steady and continuous care well into adulthood.
